# AI-Enabled Digital Phenotyping for Personalized Risk Stratification in Internet Gaming Disorder: A Privacy-Preserving Simulation Study

**DOI:** 10.3390/jpm16090447

**Published:** 2026-08-27

**Authors:** Athanasios Kranas, Evgenia Paxinou, Ioannis Bazakidis, Christina Koufopoulou, Petros Koufopoulos, Georgios Feretzakis, Vassilios S. Verykios

**Affiliations:** 1 School of Science and Technology, Hellenic Open University, 26335 Patras, Greece; paxinou.evgenia@ac.eap.gr (E.P.); georgios.feretzakis@ac.eap.gr (G.F.); verykios@eap.gr (V.S.V.); 2 School of Engineering, College of Science and Engineering, University of Edinburgh, King’s Buildings, Edinburgh EH9 3FB, UK; s2428886@ed.ac.uk; 3 Intensive Care Unit, General Hospital of Athens “KAT”, Nikis 2, 14561 Athens, Greece; chriskoufopoulou@gmail.com; 4 Cardiology Department, Sismanogleio General Hospital, Sismanogleiou 1, 15126 Athens, Greece; petroskouf@med.uoa.gr

**Keywords:** internet gaming disorder, gaming disorder, privacy-preserving digital phenotyping, behavioral telemetry, artificial intelligence, machine learning, personalized medicine, risk stratification, synthetic data, explainable AI

## Abstract

**Background/Objectives**: Assessment of Internet Gaming Disorder (IGD) relies on retrospective self-reports and clinical interviews, which may be affected by recall and social desirability biases and may be insensitive to behavioral change. This study evaluated an artificial intelligence (AI)-enabled, privacy-preserving digital phenotyping framework for personalized IGD risk stratification under controlled simulation assumptions. **Methods**: A synthetic dataset of 1000 virtual user profiles was generated with a 20% elevated-risk prevalence and 5% balanced stochastic label noise. Four aggregated telemetry features were modeled: average session duration, sessions per week, Late-Night Index, and application-switching rate. Random Forest, Logistic Regression, and Gradient Boosting classifiers were evaluated using a stratified 80:20 hold-out split, five-fold cross-validation, playtime-only baselines, label-noise sensitivity analysis, and 200 synthetic realizations. **Results**: The primary Random Forest model achieved a balanced accuracy of 0.850, a sensitivity of 0.800, a specificity of 0.900, an area under the receiver operating characteristic curve (ROC-AUC) of 0.909, an average precision (AP) of 0.779, and a Brier score of 0.089. As an internal consistency check under the pre-specified synthetic signal structure, all-feature models showed higher performance than playtime-only baselines, and feature importance analyses recovered the encoded signal hierarchy. Performance declined with increasing label noise. Across 200 realizations, mean ROC-AUC values for the three all-feature models ranged from 0.888 to 0.904, with overlapping empirical 95% intervals. **Conclusions**: The framework demonstrates the methodological feasibility of transforming aggregated telemetry into interpretable risk signals while avoiding content-level monitoring. These findings are hypothesis-generating and do not establish clinical validity or diagnostic performance. Longitudinal validation in clinically characterized cohorts is required before deployment.

## 1. Introduction

Digital gaming has become a widespread form of entertainment, social interaction, and competition across age groups. For most individuals, gaming is recreational and may be associated with social, cognitive, or motivational benefits. However, a smaller subgroup of players develops persistent and impairing patterns of gaming behavior characterized by impaired control, increasing priority given to gaming over other activities, and continuation despite negative consequences. Reflecting this clinical relevance, Internet Gaming Disorder (IGD) is included in the Diagnostic and Statistical Manual of Mental Disorders, Fifth Edition, Text Revision (DSM-5-TR) as a condition for further study, while Gaming Disorder is formally recognized in the International Classification of Diseases, Eleventh Revision (ICD-11) [1,2]. Epidemiological evidence suggests that Gaming Disorder affects only a minority of players, highlighting the need to distinguish clinically relevant dysregulation from high but non-problematic engagement [3]. Contextual factors may also influence gaming behavior. For example, a Greek case study conducted during the COVID-19 lockdown reported that 4.7% of participants met criteria consistent with internet gaming addiction and that the proportion of participants reporting more than four hours of gaming per day increased during lockdown, illustrating how social restriction and psychological stress may intensify gaming activity in vulnerable subgroups [4].

Despite growing clinical and research interest, the assessment of problematic gaming remains challenging. Current approaches rely largely on clinical interviews and retrospective self-report instruments, such as the Internet Gaming Disorder Scale–Short Form (IGDS9-SF) [5]. These tools remain important for structured assessment, but they are also limited by recall bias, social desirability bias, and limited sensitivity to dynamic changes in behavior over time. In digital media research more broadly, self-reported estimates of screen use often diverge from objectively logged behavior, raising concerns about the exclusive reliance on retrospective self-report measures [6]. This limitation is especially relevant for personalized mental health care, where risk may fluctuate across days, contexts, sleep patterns, stress levels, and individual routines.

Digital phenotyping offers a potential way to complement traditional assessment by quantifying behavior in situ through data generated during everyday interaction with digital devices [7]. In mental health research, passive sensing and behavioral telemetry have been proposed as tools for identifying person-specific patterns that may support earlier detection, longitudinal monitoring, and more individualized care pathways [8]. In the context of IGD, potentially informative signals may include not only total gaming duration, but also temporal regularity, late-night activity, session fragmentation, and interaction dynamics. Such features may help distinguish high recreational engagement from behavioral patterns that are more consistent with dysregulation, sleep disruption, attentional preoccupation, or difficulty disengaging.

However, the use of passive telemetry in mental health also raises substantial ethical, privacy, and governance concerns. Continuous monitoring can become intrusive if it involves raw keystrokes, screen capture, message content, browsing content, or other sensitive forms of personal data. For this reason, privacy-preserving approaches are essential if digital phenotyping is to become acceptable in personalized mental health care. A clinically plausible system should follow data-minimization principles, prioritize local or client-side processing where possible, and restrict analysis to aggregated behavioral indicators that are proportionate to the intended screening or monitoring purpose [9]. This is particularly important in Gaming Disorder research, where inappropriate measurement or interpretation may contribute to the over-pathologization of normal or non-problematic gaming behavior [10].

Recent empirical work has begun to explore digital phenotypes for the early detection of IGD using passive sensor data, supporting the broader feasibility of behavioral digital markers in this field [11]. In parallel, recent work has begun to examine the potential role of large language models (LLMs) in IGD assessment and care. Kranas and Verykios argued that LLMs may support prevention, assessment, treatment support, and research in IGD, but emphasized that current evidence remains preliminary, that no IGD-specific LLM therapeutic trials have yet established efficacy, and that privacy, governance, bias, and safety remain central concerns. These observations reinforce the need for transparent, privacy-preserving, and clinically cautious artificial intelligence (AI) frameworks in IGD research [12]. Nevertheless, there remains a need for transparent methodological frameworks that specifically address privacy-preserving feature extraction, interpretable machine learning, and personalized risk stratification. In this early-stage methodological context, simulation complements existing empirical work [11] by providing a transparent and reproducible test environment in which the data-generating assumptions and label uncertainty are explicitly known, while avoiding collection of sensitive real-world behavioral telemetry.

Machine learning may be useful in this context because behavioral risk patterns are unlikely to be captured by any single variable, such as total hours played. Instead, risk may emerge from combinations of temporal, interactional, and contextual features. Theoretical models of addictive behaviors, including the Interaction of Person-Affect-Cognition-Execution (I-PACE) model, emphasize that dysregulated digital behavior involves interactions among affective, cognitive, executive control, and contextual processes rather than simple exposure duration alone [13]. Interpretable AI models may therefore help identify candidate behavioral signatures while maintaining transparency about which features contribute to risk estimates. At the same time, AI-based systems in mental health must be framed cautiously: they should support screening, monitoring, and clinical decision-making rather than function as autonomous diagnostic tools.

The present study proposes a privacy-preserving, AI-enabled digital phenotyping framework for personalized risk stratification in IGD. As an early-stage methodological proof of concept, we use a reproducible synthetic dataset of virtual user profiles to evaluate whether aggregated interactional and temporal telemetry features can be transformed into interpretable risk signals under controlled simulation assumptions. The framework focuses on four candidate features: average session duration, sessions per week, Late-Night Index, and application-switching rate. Multiple machine learning models are evaluated, including Random Forest, Logistic Regression, and Gradient Boosting classifiers, with playtime-only models used as baselines.

The aim of this study is not to establish clinical validity or deployable diagnostic performance. Rather, the objective is to demonstrate the methodological feasibility of a privacy-preserving analytic pipeline and to generate hypotheses about which types of behavioral telemetry may be most relevant for future real-world validation. Specifically, this study aims to: (i) describe a non-intrusive architecture for privacy-preserving behavioral telemetry extraction; (ii) evaluate, in simulation, whether aggregated interactional and temporal features can support elevated-risk versus lower-risk stratification; (iii) compare all-feature models with playtime-only baselines; (iv) examine whether complementary explainability analyses recover the known signal hierarchy encoded in the synthetic data-generating process; and (v) assess robustness to stochastic label noise and variation across independent synthetic realizations. By positioning passive telemetry as a complementary layer rather than a replacement for clinical assessment, this work contributes to the development of privacy-aware, interpretable, and personalized approaches to digital mental health.

## 2. Materials and Methods

This section describes the simulation design, privacy-preserving telemetry architecture, candidate behavioral features, synthetic data generation procedure, machine learning models, evaluation metrics, explainability analyses, and robustness checks. Because the study used only synthetic virtual user profiles, the methodological framework was designed to support reproducibility, interpretability, and cautious simulation-based inference rather than clinical validation.

### 2.1. Study Design

This study was designed as a simulation-based methodological proof of concept for privacy-preserving, AI-enabled digital phenotyping in Internet Gaming Disorder (IGD). The aim was not to validate a diagnostic model or estimate real-world clinical performance, but to evaluate whether aggregated temporal and interactional telemetry features could be transformed into interpretable risk stratification signals under controlled simulation assumptions.

**Figure 1:**
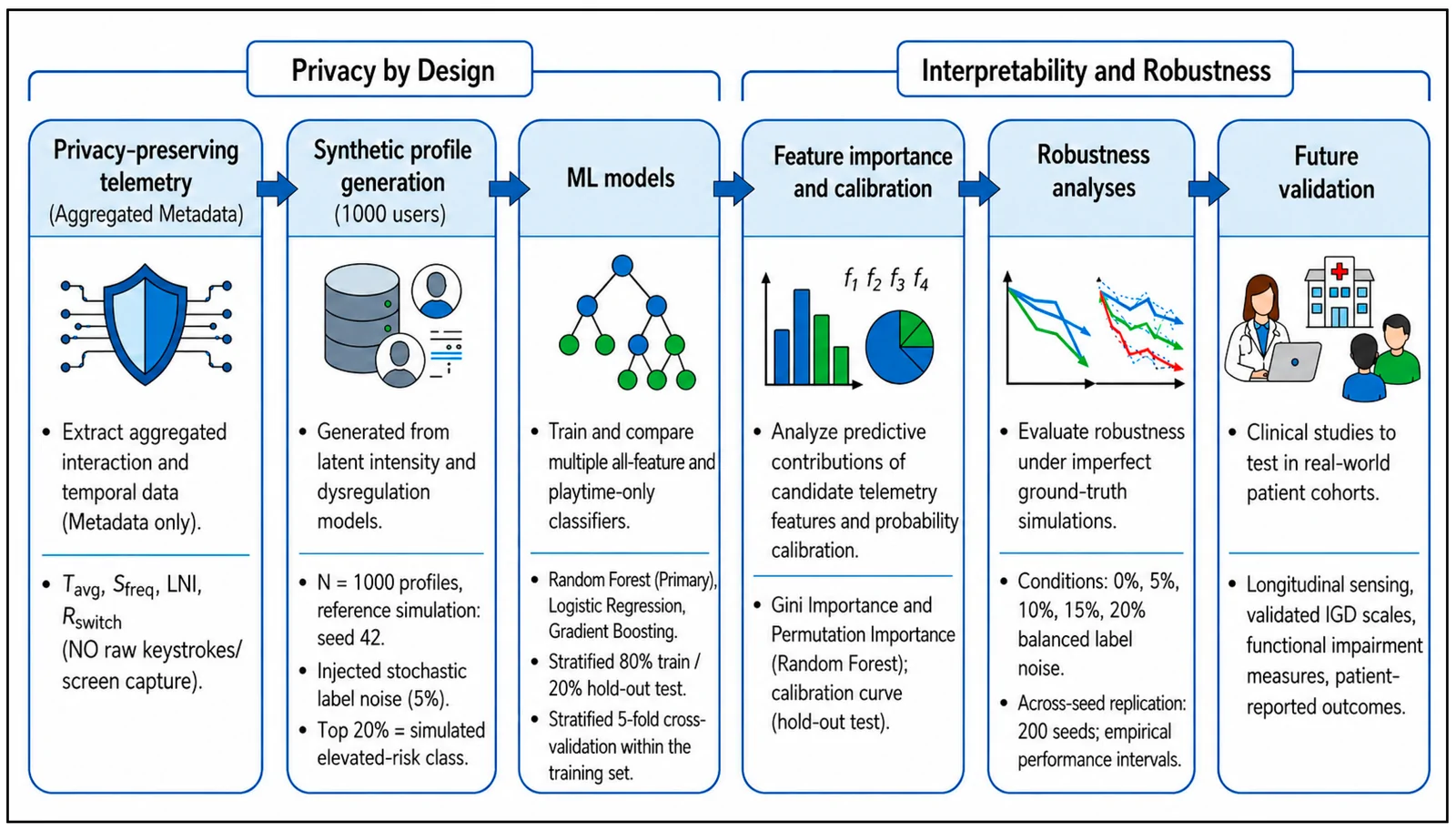
Methodological workflow of the proposed privacy-preserving, AI-enabled digital phenotyping simulation pipeline. Aggregated metadata are used to generate synthetic user profiles, train and compare machine learning models, evaluate performance using hold-out testing and stratified cross-validation, examine feature importance and calibration, assess robustness across label-noise conditions and independent simulation seeds, and define requirements for future validation in real-world clinically characterized cohorts.

The reference synthetic dataset of 1000 virtual user profiles was generated using random seed 42. The simulation was used to test the feasibility of the proposed analytic pipeline while avoiding the ethical and privacy challenges associated with continuous system-level monitoring of real users. The overall workflow consisted of (i) privacy-preserving feature specification; (ii) synthetic user profile generation; (iii) stochastic label-noise injection; (iv) supervised machine learning classification; (v) comparative hold-out evaluation against baseline models; (vi) stratified five-fold cross-validation within the training set; (vii) model inspection, including feature importance and calibration analyses; and (viii) robustness analyses across label-noise conditions and independent simulation seeds. The overall methodological workflow of the proposed privacy-preserving digital phenotyping pipeline is summarized in Figure 1.

Because no real human participants, patient records, or identifiable behavioral logs were used, the present study should be interpreted as a computational simulation rather than a clinical validation study.

### 2.2. Privacy-Preserving Digital Phenotyping Architecture

The proposed system architecture is conceptualized as a lightweight client-side telemetry layer that extracts only aggregated temporal and interactional metadata from gaming-related activity. The architecture follows a Privacy-by-Design approach by excluding raw keystroke content, screenshots, screen recordings, message content, browsing content, voice data, and other content-level information. This design is aligned with the Privacy-by-Design principle that privacy safeguards should be embedded into the technical architecture of a system from the outset rather than added retrospectively [14].

Accordingly, the framework restricts data capture to low-dimensional behavioral summaries that are relevant to risk stratification while minimizing the collection of sensitive personal information. All primary feature extraction is designed to occur locally at the client level before any risk score is generated. In a real-world implementation, such a design would allow the system to transmit only derived behavioral indicators or risk summaries rather than raw behavioral streams.

This privacy-preserving structure is important because behavioral telemetry in mental health may reveal sensitive information even when content-level data are not collected. Therefore, the proposed architecture emphasizes data minimization, proportionality, local processing, transparency, and clear separation between behavioral monitoring and clinical diagnosis.

### 2.3. Candidate Behavioral Telemetry Features

Four candidate features were specified to represent temporal and interactional aspects of gaming behavior. These features were selected because they are interpretable, technically feasible to derive from aggregated metadata, and theoretically relevant to behavioral dysregulation. Table 1 summarizes the four candidate telemetry features, their notation, operational definitions, and theoretical relevance.

Each virtual user i was represented by the feature vector:

(1) $$X_{i}=\left[f_{1i},f_{2i},f_{3i},f_{4i}\right]$$

where $f_{1i}$ to $f_{4i}$ corresponded to the following telemetry indicators:

(2) $$f_{1i}=T_{avg,i}$$

where $T_{avg,i}$ is the average duration of uninterrupted gaming sessions, expressed in hours.

(3) $$f_{2i}=S_{freq,i}$$

where $S_{freq,i}$ is the number of gaming sessions initiated within a seven-day period.

(4) $$f_{3i}={\mathrm{LNI}}_{i}=\frac{T_{night}}{T_{total}}$$

where ${\mathrm{LNI}}_{i}$ is the Late-Night Index, defined as the proportion of total gaming time occurring between 00:00 and 06:00.

(5) $$f_{4i}=R_{switch,i}=\frac{N_{switch}}{T_{active}}$$

where $R_{switch,i}$ is the application-switching rate, defined as the number of application-switching events per active gaming hour.

The rationale for including $T_{avg,i}$ and $S_{freq,i}$ was to capture general engagement intensity. However, these variables were not assumed to be sufficient indicators of a disorder, because high gaming duration or frequency may also reflect recreational, social, or competitive engagement. The ${\mathrm{LNI}}_{i}$ feature was included to capture potential circadian disruption and the persistence of gaming during sleep-relevant hours. The $R_{switch,i}$ feature was included as an exploratory interactional marker that may reflect fragmented attention, repeated task switching, or difficulty disengaging from gaming-related activity. The broader rationale for these candidate constructs was informed by empirical digital phenotyping work and theoretical models of addictive behavior [11,13]; however, the specific telemetry features should not be regarded as established IGD biomarkers and require independent empirical validation.

### 2.4. Synthetic Data Generation

A reproducible synthetic dataset of 1000 virtual user profiles was generated to represent plausible variation in gaming-related behavioral patterns. The purpose of the simulation was not to reproduce the full complexity of real gaming behavior, but to create a transparent and controlled test environment for evaluating the proposed privacy-preserving analytic pipeline.

Two unobserved latent variables were generated for each virtual user: latent gaming intensity and latent behavioral dysregulation. These latent variables were sampled from standard normal distributions and were used only to generate correlated behavioral patterns. They were not included as predictors in the machine learning models.

(6) $$I_{i}∼N\left(0,1\right)$$

(7) $$D_{i}∼N\left(0,1\right)$$

where $I_{i}$ denotes latent gaming intensity and $D_{i}$ denotes latent behavioral dysregulation for virtual user i.

Four observable telemetry features were then generated from these latent variables. Average session duration and sessions per week were designed to reflect general engagement intensity, whereas the Late-Night Index and application-switching rate were designed to represent temporal and interactional dysregulation. Values were bounded to avoid implausible observations, such as negative session durations or proportions outside the interval $\left[0,1\right]$.

Average session duration was generated as:

(8) $$T_{avg,i}=clip\left(1.40+0.75I_{i}+0.25D_{i}+\epsilon_{1i},\;0.20,\;7.50\right)$$

where $\epsilon_{1i}∼N\left(0,0.65^{2}\right)$.

Sessions per week were generated as:

(9) $$S_{freq,i}=clip\left[round\left(4.50+2.10I_{i}+0.65D_{i}+\epsilon_{2i},\;1,\;21\right)\right]$$

where $\epsilon_{2i}∼N\left(0,1.80^{2}\right)$.

The Late-Night Index was generated as:

(10) $$LNI_{i}=clip\left[\sigma \left(-2.30+0.35I_{i}+1.25D_{i}+\epsilon_{3i}\right),0,1\right]$$

where $\epsilon_{3i}∼N\left(0,0.80^{2}\right)$ and $\sigma \left(x\right)$ is the logistic sigmoid function.

The application-switching rate was generated as:

(11) $$R_{switch,i}=clip\left[exp\left(0.60+0.15I_{i}+0.55D_{i}+\epsilon_{4i}\right),\;0.20,\;12.00\right]$$

where $\epsilon_{4i}∼N\left(0,0.35^{2}\right)$.

The four observed features were standardized and used to calculate a continuous latent risk score:

(12) $$Risk_{i}\;=\;0.15z\left(T_{avg,i}\right)\;+\;0.05z\left(S_{freq,i}\right)\;+\;0.85z\left(LNI_{i}\right)\;+\;1.05z\left(R_{switch,i}\right)\;+\;\eta_{i}$$

where $\eta_{i}∼N\left({0,0.75}^{2}\right)$. Larger weights were deliberately assigned to the Late-Night Index and application-switching rate than to average session duration or sessions per week to define a dysregulation-dominant synthetic signal structure. Consequently, the all-feature versus playtime-only comparison was interpreted as an internal consistency check of the imposed simulation structure rather than as an empirical test of the relative value of these features. The $z\left(\cdot \right)$ transformation was computed across all 1000 synthetic profiles during the data generation stage to define the simulated latent risk score; it was not a model-preprocessing step fitted during evaluation. The additive noise term $\eta_{i}∼N\left({0,0.75}^{2}\right)$ was included as an irreducible simulation noise floor, meaning that the clean-label condition was not expected to be perfectly separable even when stochastic label flipping was set to 0%. The full synthetic data generation parameterization is provided in Supplementary Table S1, and the stepwise procedure is described in Algorithm A1.

The top 20% of virtual users according to the latent risk score were assigned to the clean elevated-risk class. To approximate imperfect ground truth, a 5% balanced stochastic label noise was then introduced by flipping equal numbers of positive and negative labels. Specifically, 25 clean elevated-risk labels were changed to lower-risk labels and 25 clean lower-risk labels were changed to elevated-risk labels. This preserved the elevated-risk prevalence at 20%.

The final binary outcome was coded as 0 for lower-risk and 1 for elevated-risk. This outcome represents a simulated elevated-risk status and should not be interpreted as clinically diagnosed IGD.

### 2.5. Machine Learning Models

A supervised binary classification approach was used to estimate simulated elevated-risk status from the candidate telemetry features. The Random Forest classifier was selected as the primary illustrative model because tree-based ensemble methods can model nonlinear feature relationships and interactions while also providing intuitive feature importance estimates [15].

The Random Forest classifier was implemented in Python (version 3.13.15) using the scikit-learn machine learning library (version 1.6.1) [16]. The model was specified with 100 decision trees, a maximum depth of 5, a fixed random seed of 42, and class weighting set to balanced. The constrained maximum depth was used to reduce the likelihood of overfitting to simulation noise and to preserve interpretability.

To assess whether model behavior was consistent across classifier families, two additional all-feature classifiers were also trained: Logistic Regression and Gradient Boosting [17]. Logistic Regression was implemented using a scikit-learn pipeline in which the StandardScaler was fitted only on the training data and subsequently applied to the hold-out test data. The Logistic Regression classifier used maximum iterations set to 1000, class weighting set to balanced, and a fixed random seed of 42. Gradient Boosting was implemented with 100 estimators, a maximum tree depth of 3, and a fixed random seed of 42. The evaluated model families, feature sets, and comparison purposes are summarized in Supplementary Table S2.

Random Forest was selected a priori as the primary illustrative model, before examination of the comparative model results, because it can represent nonlinear relationships while providing direct feature importance measures. Logistic Regression and Gradient Boosting were included as linear and nonlinear comparators, respectively. Model configurations were fixed for this methodological proof of concept; no data-driven hyperparameter optimization was performed, and the hold-out test set was not used for model tuning.

Two playtime-only baseline models were also evaluated. These models used only average session duration $T_{avg,i}$ as the predictor and were implemented using Random Forest and Logistic Regression. Because average session duration carried a comparatively small pre-specified weight in the latent risk function, these baselines were used as internal consistency comparators rather than as empirical tests of whether multivariate telemetry is superior to playtime in real-world IGD.

The dataset was divided into training and testing sets using scikit-learn’s train_test_split function with an 80:20 split, stratify = y, and random_state = 42. Stratification preserved the lower-risk and elevated-risk class distribution across the training and test sets. The final test set included 200 virtual profiles, with 160 lower-risk profiles and 40 elevated-risk profiles.

Within the 80% training set, stratified five-fold cross-validation was additionally performed using five shuffled folds with random_state = 42 to characterize development set model performance. The same pre-specified model configurations were used, and the independent 20% hold-out test set remained excluded from this procedure.

The z-standardization used to construct the synthetic latent risk score occurred only during data generation and was not a model-preprocessing step; predictor scaling during model evaluation was applied only within the Logistic Regression pipeline.

### 2.6. Model Evaluation

Model performance was evaluated on the unseen 20% hold-out test set. Because the purpose of the model was risk stratification rather than diagnosis, performance was assessed using multiple complementary metrics rather than overall accuracy alone.

The following metrics were calculated: overall accuracy, balanced accuracy, sensitivity/recall for the elevated-risk class, specificity for the lower-risk class, precision/positive predictive value, negative predictive value, F1-score, the area under the receiver operating characteristic curve, average precision, the Brier score, and the confusion matrix. Discrimination was assessed using ROC-AUC, while precision–recall performance was summarized using average precision (AP) [18,19]. The Brier score was included as a measure of overall probabilistic prediction performance, calculated from the mean squared difference between predicted probabilities and observed outcomes [20]. For the primary Random Forest model, calibration was additionally examined using a 10-bin calibration curve on the hold-out test set; no post hoc probability recalibration was applied.

Accuracy was retained for comparability with standard classification reporting, but greater emphasis was placed on sensitivity, specificity, precision, discrimination, and calibration because these metrics are more informative for potential screening support applications. In a future clinical setting, false positives could contribute to over-pathologization of high recreational engagement, whereas false negatives could fail to identify users who may require further assessment. Therefore, the evaluation was interpreted as a simulation-based assessment of methodological feasibility rather than evidence of clinical performance.

### 2.7. Feature Importance and Explainability Analysis

Model interpretability was examined using two complementary feature importance methods applied to the primary Random Forest model. Because the simulated outcome was generated from a known weighted latent risk function, feature importance analysis was interpreted as a positive-control analysis rather than as empirical marker discovery. The aim was to evaluate whether the model inspection procedures recovered the known signal hierarchy encoded in the synthetic data-generating process.

First, impurity-based Random Forest feature importance was used to estimate the contribution of each telemetry feature to the model’s classification decisions. Second, permutation feature importance was evaluated on the hold-out test set using ROC-AUC as the scoring metric. Permutation importance was calculated using 50 repeated permutations for each feature. This complementary analysis was included because impurity-based feature importance can be sensitive to feature scale, distribution, and correlation structure, whereas permutation importance evaluates the decrease in predictive performance after feature perturbation on held-out data [15,21]. Pairwise Pearson correlations among the four generated telemetry features were calculated to characterize the covariance structure relevant to the interpretation of the feature importance analyses.

Feature importance results were interpreted as simulation-derived indicators of internal consistency within the synthetic framework. They were not interpreted as validated clinical biomarkers or empirically discovered IGD predictors. The purpose of the feature importance analysis was therefore to assess whether the pipeline behaved consistently with the known synthetic data-generating structure, not to establish clinically valid digital markers. No data-driven feature selection was performed; all four candidate telemetry features were pre-specified and retained in each all-feature model.

### 2.8. Sensitivity and Robustness Analyses

To evaluate robustness to imperfect ground truth—a key simulation assumption and a known challenge in supervised classification with noisy labels—sensitivity analyses were conducted across different levels of stochastic label noise [22]. Specifically, the Random Forest model was re-estimated under label-noise conditions of 0%, 5%, 10%, 15%, and 20%. The same label-noise sensitivity analysis was additionally applied to Logistic Regression and Gradient Boosting to assess whether the observed degradation pattern was specific to Random Forest.

For each label-noise condition, the same synthetic data generation procedure was applied using the fixed random seed of 42. Balanced label flipping was used so that the elevated-risk prevalence remained 20% across all conditions. Model performance was then compared across label-noise levels using the same evaluation metrics described above. Feature-ranking stability was also examined by identifying the top feature according to impurity-based Random Forest importance and permutation importance within each label-noise condition.

This robustness analysis was important because the current study relies on simulated labels rather than clinically confirmed IGD diagnoses. Stable feature rankings across increasing label-noise conditions would support the methodological robustness of the proposed pipeline under imperfect measurement conditions. Conversely, unstable rankings would indicate that the findings are highly dependent on the assumptions used during synthetic data generation.

To assess sensitivity to the particular synthetic realization, the primary 5% label-noise data generation and hold-out model-evaluation procedure was additionally repeated across 200 random seeds. Performance distributions were summarized using the mean, standard deviation, and empirical 2.5th and 97.5th percentiles. The label-noise sensitivity analysis held the underlying seed-42 feature realization constant while varying label noise, whereas the across-seed analysis held the primary 5% label-noise condition constant to characterize variability across synthetic realizations. For each realization in the across-seed analysis, the corresponding seed was used for synthetic data generation, the stratified train–test split, and stochastic model components.

### 2.9. Reporting and Reproducibility

The study was reported as a simulation-based proof of concept and not as a clinical prediction-model validation study. Nevertheless, relevant principles from prediction-model reporting guidance, including transparent description of predictors, outcome definition, model specification, evaluation procedures, and reproducibility, were followed where applicable [23].

All synthetic data generation parameters, model specifications, train–test split settings, label-noise conditions, and evaluation metrics are reported in this manuscript to support reproducibility. The complete Python analysis code, reference seed-42 synthetic dataset, and analysis outputs supporting all reported analyses are openly available in Zenodo at https://doi.org/10.5281/zenodo.22018420. Because all records are synthetically generated, the repository contains no real participant data, patient records, identifiable behavioral logs, or sensitive personal information. Detailed pseudocode for the synthetic data generation procedure and the model-evaluation pipeline is provided in Algorithms A1 and A2, respectively, in Appendix A.

## 3. Results

This section reports the synthetic cohort composition, primary Random Forest performance, comparative and cross-validated model results, calibration, feature importance analyses, and sensitivity and robustness analyses. All findings refer to simulated elevated-risk status and should therefore be interpreted as proof-of-concept evidence of methodological feasibility rather than as estimates of real-world clinical performance.

### 3.1. Synthetic Cohort and Evaluation Sample

The reproducible simulation generated 1000 virtual user profiles. After balanced stochastic label-noise injection, 800 profiles were assigned to the lower-risk class and 200 profiles were assigned to the elevated-risk class, corresponding to an elevated-risk prevalence of 20%. The dataset was divided into training and test sets using an 80:20 stratified split. The hold-out test set included 200 virtual profiles, consisting of 160 lower-risk profiles and 40 elevated-risk profiles.

Unless otherwise specified, the seed 42 point estimates reported below refer to performance on the independent hold-out test set; cross-validation and across-seed results are explicitly identified where reported. Because the dataset was synthetically generated, the results should be interpreted as simulation-based evidence of methodological feasibility rather than as clinical validation or deployable diagnostic performance.

### 3.2. Primary Random Forest Classification Performance

The primary Random Forest classifier was trained using all four candidate telemetry features: average session duration, sessions per week, the Late-Night Index, and the application-switching rate. On the hold-out test set, the model achieved an overall accuracy of 0.880 and a balanced accuracy of 0.850. Sensitivity for the elevated-risk class was 0.800, indicating that the model correctly identified 80.0% of simulated elevated-risk profiles. Specificity was 0.900, indicating that 90.0% of lower-risk profiles were correctly classified.

**Figure 2:**
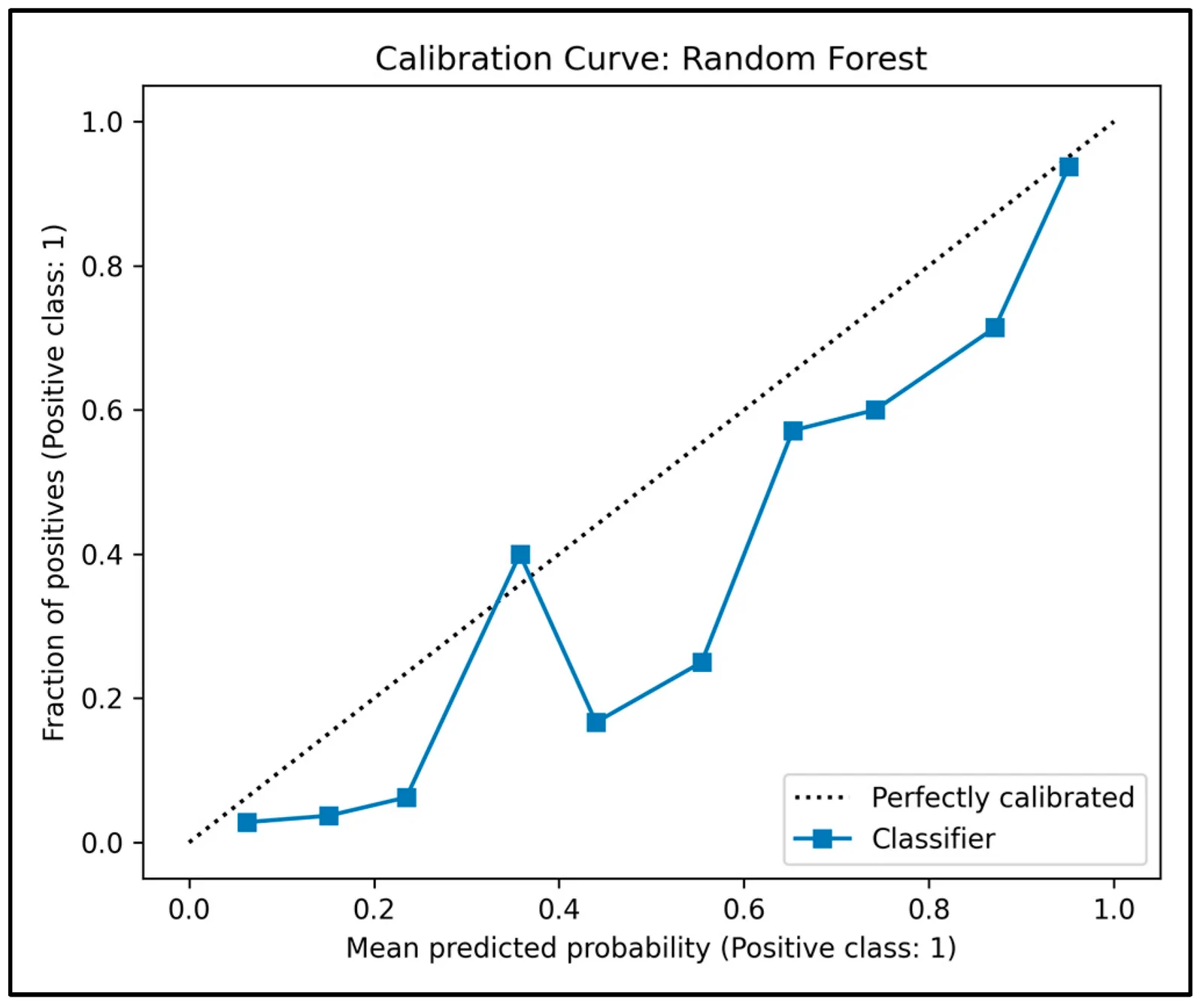
Calibration curve for the primary Random Forest model on the hold-out test set. The dashed diagonal represents perfect calibration; observed event fractions are plotted against mean predicted probabilities across 10 probability bins.

Precision for the elevated-risk class was 0.667, while the negative predictive value was 0.947. The F1-score for the elevated-risk class was 0.727. Discrimination performance was strong, with an ROC-AUC of 0.909 and an AP of 0.779. For context, with an elevated-risk prevalence of 20%, the no-skill reference for average precision is 0.20. The Brier score was 0.089. The corresponding calibration curve showed departures from perfect calibration, particularly across intermediate predicted probability ranges (Figure 2).

The confusion matrix showed 144 true negatives, 16 false positives, 8 false negatives, and 32 true positives. Thus, the model showed stronger performance in ruling out lower-risk profiles than in confirming elevated-risk profiles, as reflected by the higher negative predictive value compared with the positive predictive value. This pattern is consistent with the intended role of the proposed framework as a screening support and risk stratification approach rather than as a standalone diagnostic system. Detailed metrics for the primary Random Forest model, including confusion-matrix counts, are provided in Supplementary Table S3.

### 3.3. Comparative Model Performance and Playtime-Only Baselines

To evaluate whether the fitted models recovered the imposed multivariate signal structure, the primary Random Forest model was compared with alternative all-feature classifiers and playtime-only baseline models. As expected under the pre-specified synthetic signal structure, the all-feature models showed higher performance than the playtime-only models across most discrimination and classification metrics.

Among the all-feature models, Gradient Boosting achieved the highest overall accuracy (0.900), specificity (0.962), and precision (0.812), and the lowest Brier score (0.073), but it showed lower sensitivity for the elevated-risk class (0.650). Logistic Regression achieved the highest sensitivity (0.850), ROC-AUC (0.920), and AP (0.832), indicating strong discrimination despite its simpler linear structure. The Random Forest model showed a balanced performance profile, with an accuracy of 0.880, a sensitivity of 0.800, a specificity of 0.900, an ROC-AUC of 0.909, and an AP of 0.779.

As expected under the pre-specified synthetic signal structure, the playtime-only models showed lower discrimination than the all-feature models. The Random Forest model using only average session duration achieved an ROC-AUC of 0.686 and an AP of 0.497, while the Logistic Regression playtime-only model achieved an ROC-AUC of 0.724 and an AP of 0.547.

Within the training set, stratified five-fold cross-validation yielded mean ± SD ROC-AUC values of 0.880 ± 0.027 for Random Forest, 0.894 ± 0.027 for Logistic Regression, and 0.860 ± 0.040 for Gradient Boosting. Complete cross-validation performance for all five evaluated models is presented in Table 2, with metrics summarized as the mean ± SD across the five validation folds. The corresponding cross-validation summary is also provided in Supplementary Table S4.

The comparison provides an internal consistency check, showing that the models recover the multivariate signal structure imposed by the synthetic data-generating process. The higher ROC-AUC and AP of the all-feature models should therefore be interpreted within the assumptions of the simulation, rather than as empirical evidence that these features are more informative than playtime in real-world IGD. Table 3 summarizes the comparative performance of the all-feature models and playtime-only baseline models on the hold-out test set.

**Figure 3:**
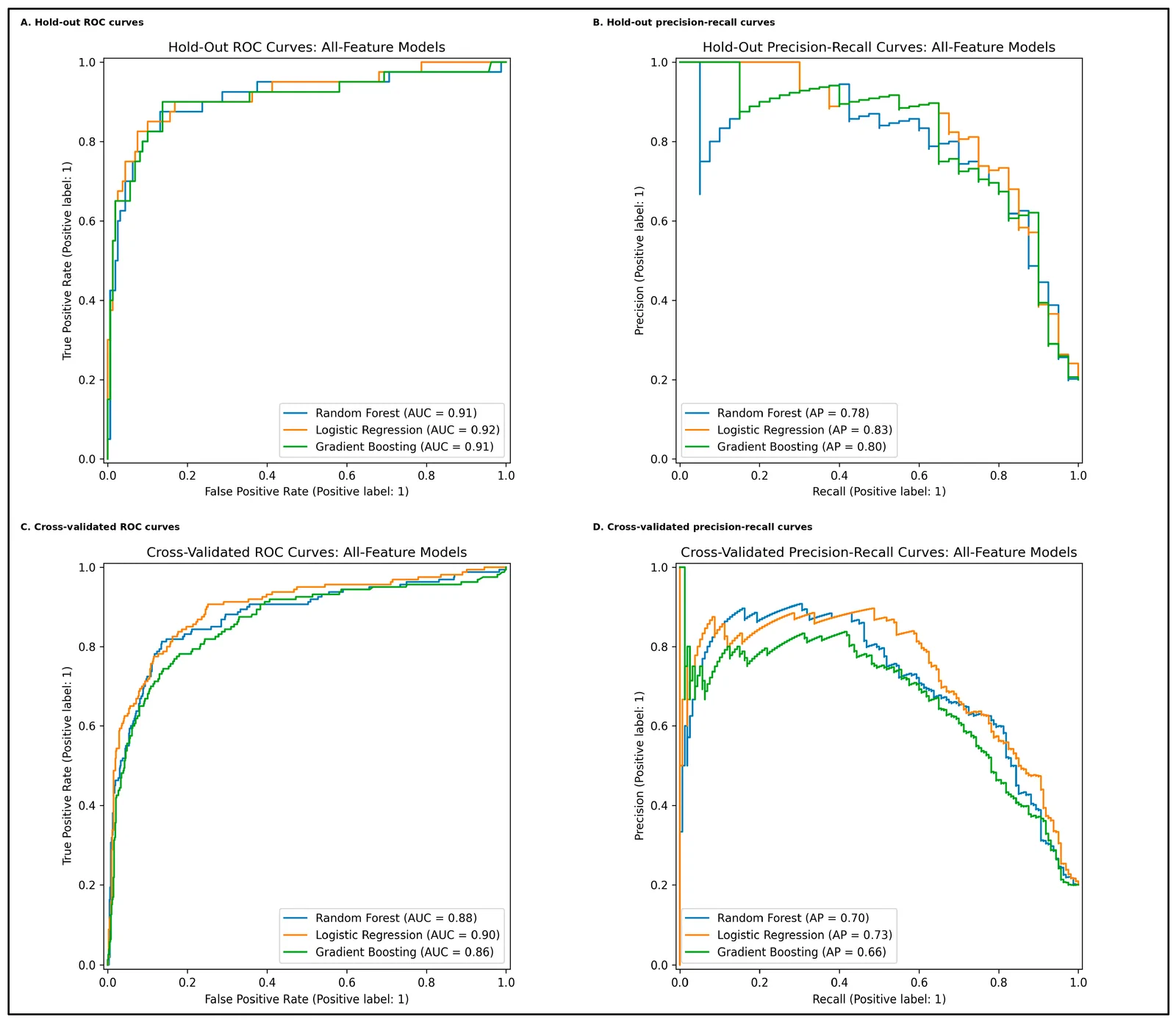
Discrimination performance of the all-feature models. (**A**) Receiver operating characteristic (ROC) curves for Random Forest, Logistic Regression, and Gradient Boosting on the independent hold-out test set; (**B**) corresponding precision–recall curves on the hold-out test set; (**C**) ROC curves based on out-of-fold predictions from stratified five-fold cross-validation within the training set; and (**D**) corresponding cross-validated precision–recall curves. Values shown in panels (**C**,**D**) are calculated from pooled out-of-fold predictions and may therefore differ slightly from the mean fold-specific metrics reported in Table 2.

Comparative ROC and precision–recall curves for the three all-feature models on the hold-out test set and under stratified five-fold cross-validation are shown in Figure 3.

### 3.4. Feature Importance and Explainability Results

Feature importance analysis was conducted as a positive-control analysis to examine whether the model inspection procedures recovered the known hierarchy of signal strength encoded in the synthetic data-generating process. In the latent risk score, the application-switching rate and the Late-Night Index were assigned the largest weights, followed by average session duration and sessions per week. Therefore, the feature importance analysis was not intended to discover empirical IGD markers, but to evaluate whether the proposed pipeline behaved consistently with the known simulation structure.

Impurity-based Random Forest importance recovered the expected ordering of simulated signal strength. Application-switching rate, which had the largest pre-specified weight in the latent risk-score function, showed the highest Gini-based importance value, accounting for 44.36% of total feature importance. The Late-Night Index, which had the second-largest pre-specified weight, ranked second and accounted for 40.52%. Average session duration contributed 12.21%, while sessions per week contributed 2.91%. Together, the application-switching rate and the Late-Night Index accounted for approximately 85% of the total impurity-based importance, consistent with the pre-specified latent risk-score structure.

Permutation importance produced a similar feature hierarchy. The application-switching rate had the largest mean decrease in ROC-AUC when permuted, with a mean importance of 0.0877. The Late-Night Index ranked second, with a mean importance of 0.0806. Average session duration showed a smaller contribution, with a mean importance of 0.0273, while sessions per week had near-zero permutation importance, with a mean value of −0.0008. The near-zero or slightly negative permutation value for sessions per week is consistent with its small pre-specified contribution to the latent risk function and should not be interpreted as evidence of limited importance in real-world IGD.

The generated features were correlated as expected from their shared latent structure. The strongest correlations were between the Late-Night Index and the application-switching rate (r = 0.672) and between average session duration and sessions per week (r = 0.596); the remaining pairwise correlations ranged from 0.282 to 0.329 (Supplementary Table S7). Because correlated predictors can share predictive information, the importance estimates should not be interpreted as unique or causal contributions of individual features.

These results should be interpreted as evidence of internal consistency within the simulation framework. They do not demonstrate that application-switching rate or late-night gaming are validated clinical biomarkers or empirically established predictors of IGD. Rather, they show that, under the controlled assumptions of the simulation, the model and interpretability procedures recover the expected pre-specified signal hierarchy. Real-world longitudinal studies are required to test whether analogous temporal or interactional telemetry features have clinical relevance.

Table 4 reports the impurity-based and permutation-based feature importance estimates for the primary Random Forest model.

### 3.5. Sensitivity and Robustness Results

A sensitivity analysis was conducted to evaluate whether model performance remained stable under increasing levels of stochastic label noise. The Random Forest model was re-estimated under five label-noise conditions: 0%, 5%, 10%, 15%, and 20%. The elevated-risk prevalence was held constant at 20% across all conditions through balanced label flipping.

As expected, model performance declined progressively as label noise increased. In the no-noise condition, the model achieved strong performance, with an accuracy of 0.925, a balanced accuracy of 0.944, a sensitivity of 0.975, a specificity of 0.913, an ROC-AUC of 0.990, and an AP of 0.966. Under the primary 5% label-noise condition, performance remained strong, with an accuracy of 0.880, a balanced accuracy of 0.850, a sensitivity of 0.800, a specificity of 0.900, an ROC-AUC of 0.909, and an AP of 0.779.

At higher levels of label noise, performance decreased more substantially. With 10% label noise, the ROC-AUC declined to 0.816 and the AP to 0.570. With 15% label noise, ROC-AUC further decreased to 0.750 and the AP to 0.481. At 20% label noise, performance was weakest, with a balanced accuracy of 0.650, a sensitivity of 0.425, an ROC-AUC of 0.670, and an AP of 0.392. The Brier score also worsened progressively, increasing from 0.051 in the no-noise condition to 0.184 in the 20% label-noise condition.

Across all label-noise conditions, the application-switching rate remained the top feature according to impurity-based Random Forest importance. The top permutation-importance feature was also the application-switching rate in most conditions, although the Late-Night Index ranked highest under the 10% label-noise condition. Overall, the sensitivity analysis suggests that the proposed simulation pipeline behaves plausibly under increasing label uncertainty: discrimination decreases and Brier scores worsen as ground truth noise increases, while the relative importance of interactional and temporal features remains broadly stable. Table 5 summarizes Random Forest performance and feature-ranking stability across increasing label-noise conditions.

The comparator models showed the same overall degradation pattern. At 20% label noise, the ROC-AUC was 0.670 for Random Forest, 0.700 for Logistic Regression, and 0.673 for Gradient Boosting; corresponding AP values were 0.392, 0.450, and 0.391, respectively. Full results across all noise conditions are reported in Supplementary Table S5.

Across 200 independent realizations of the primary 5% label-noise condition, the mean ROC-AUC was 0.894 for Random Forest (empirical 95% interval, 0.839–0.950), 0.904 for Logistic Regression (0.835–0.956), and 0.888 for Gradient Boosting (0.821–0.944). The substantial overlap of these distributions supports cautious interpretation of the small differences observed in the seed 42 realization. Across-seed distributions for all reported performance metrics and all five evaluated models are presented in Table 6. The corresponding across-seed summary is also provided in Supplementary Table S6.

## 4. Discussion

This section interprets the simulation findings in relation to privacy-preserving digital phenotyping, personalized risk stratification, and responsible AI in mental health. The discussion emphasizes the methodological implications of the results while distinguishing simulation-derived performance from clinical validity, diagnostic accuracy, or practical deployability.

### 4.1. Principal Findings

This study presented a privacy-preserving, AI-enabled digital phenotyping framework for personalized risk stratification in Internet Gaming Disorder (IGD). Using a reproducible synthetic simulation, we evaluated whether aggregated temporal and interactional telemetry features could support simulated elevated-risk classification while avoiding content-level data capture. The main methodological finding was that the analytic pipeline recovered the pre-specified synthetic signal structure and showed predictable performance degradation as label uncertainty increased. In the primary 5% label-noise condition, the Random Forest classifier achieved an accuracy of 0.880, a balanced accuracy of 0.850, a sensitivity of 0.800, a specificity of 0.900, an ROC-AUC of 0.909, and an AP of 0.779 on the hold-out test set.

These findings should be interpreted as methodological and hypothesis-generating rather than clinical. Because the dataset was synthetically generated, the results do not establish diagnostic validity, clinical utility, or real-world deployability. Instead, they demonstrate that, under transparent simulation assumptions, privacy-preserving behavioral features can be transformed into interpretable risk signals. This supports the feasibility of the proposed analytic pipeline and provides a rationale for future empirical validation in real-world longitudinal cohorts.

### 4.2. All-Feature Versus Playtime-Only Comparison

Under the pre-specified synthetic signal structure, the playtime-only models were expected to perform less well than models using all four features. The playtime-only Random Forest model showed lower discrimination than the all-feature models, with an ROC-AUC of 0.686 and an AP of 0.497. Similarly, the playtime-only Logistic Regression model achieved an ROC-AUC of 0.724 and an AP of 0.547. In contrast, the all-feature models achieved ROC-AUC values above 0.90 and AP values between 0.779 and 0.832.

This comparison should therefore be interpreted as a check that the analytic pipeline recovers the imposed multivariate signal structure, rather than as evidence that these features are more informative than gaming duration in real-world populations. This is consistent with clinical concerns about over-pathologizing high engagement. Many individuals may spend substantial time gaming for recreational, social, competitive, or occupational reasons without experiencing clinically meaningful impairment. Therefore, risk stratification systems should not rely exclusively on exposure duration. Features such as nocturnal gaming, irregular interaction patterns, and difficulty disengaging may provide candidate signals of behavioral dysregulation, although this remains to be tested empirically.

From a personalized medical perspective, this distinction is important. A uniform threshold based on gaming duration cannot adequately capture individual differences in routines, sleep schedules, obligations, coping patterns, or functional consequences. A privacy-preserving digital phenotyping approach could instead support individualized monitoring by identifying deviations in a person’s own behavioral patterns over time. Such a system would be most useful not as a diagnostic substitute, but as a complementary layer that helps decide when further assessment may be warranted.

### 4.3. Interpretability and Candidate Digital Markers

Under the pre-specified assumptions of the synthetic data-generating process, the feature importance analyses recovered the expected ordering of simulated signal strength. Application-switching rate and Late-Night Index, which were assigned the largest weights in the latent risk score, also showed the largest impurity-based and permutation-importance values. Average session duration showed more modest importance, while sessions per week showed the lowest importance values under the fitted Random Forest model.

This result should be interpreted as a positive-control finding rather than as empirical marker discovery. In other words, the feature importance analysis demonstrates that the proposed pipeline can recover the known structure embedded in the synthetic generator. It does not show that application-switching behavior or late-night gaming are established clinical predictors of IGD in real-world populations.

Nevertheless, the pre-specified feature classes remain theoretically plausible targets for future empirical evaluation. Application-switching behavior may be conceptualized as a possible indicator of fragmented attention, repeated task shifting, or difficulty disengaging from gaming-related activity. Late-night gaming may be conceptualized as a possible indicator of circadian disruption, the persistence of gaming during sleep-relevant hours, or the displacement of restorative routines. These interpretations are conceptually compatible with models of problematic digital behavior that emphasize impaired control, preoccupation, affective regulation, and executive-function processes rather than exposure time alone [13].

Therefore, the feature hierarchy should be understood as a structured simulation assumption that is successfully recovered by the pipeline, not as evidence of validated biomarkers. Real-world studies are needed to determine whether application switching, nocturnal activity, or other interactional features predict validated IGD symptoms, functional impairment, sleep disruption, or treatment-relevant outcomes.

### 4.4. Robustness Under Label Noise and Across Synthetic Realizations

The sensitivity analysis across label-noise conditions provides an important methodological contribution. Performance declined progressively as stochastic label noise increased from 0% to 20%. In the no-noise condition, the ROC-AUC was 0.990 and the AP was 0.966. Under the primary 5% noise condition, the ROC-AUC remained strong at 0.909 and AP at 0.779. At 20% label noise, however, ROC-AUC declined to 0.670 and AP to 0.392. Across 200 synthetic realizations under the primary 5% label-noise condition, mean ROC-AUC values were 0.894 for Random Forest, 0.904 for Logistic Regression, and 0.888 for Gradient Boosting, with substantially overlapping empirical 95% intervals.

This pattern is plausible and desirable from a simulation-validity perspective. A model that remained nearly perfect under high label noise would raise concerns about circularity or unrealistic separability. Instead, the observed degradation suggests that the pipeline is sensitive to increasing uncertainty in the outcome label. This is relevant to behavioral-health research because clinical and self-report-derived labels may be affected by recall bias, social desirability, incomplete clinical information, and threshold ambiguity, thereby creating noisy outcome labels in supervised classification [6,22].

At the same time, the robustness analysis reinforces the need for caution. The model’s performance depends strongly on the quality of the target label. In real-world deployment, a telemetry-based system would require validated outcome definitions, careful calibration, and repeated external validation across populations. Inaccurate labels could lead to biased or unstable models, particularly when distinguishing high recreational engagement from clinically impairing gaming behavior.

### 4.5. Comparison Across Algorithms

The model-comparison analysis showed that the all-feature set performed well across different algorithms under the assumptions of the synthetic simulation. Logistic Regression achieved the highest ROC-AUC and AP, while Gradient Boosting achieved the highest accuracy, specificity, and precision, and the lowest Brier score. Random Forest remained the primary illustrative model because it had been selected a priori for its nonlinear modeling capability and direct feature importance outputs, not because it achieved superior observed performance.

These algorithmic differences should be interpreted cautiously. The strong performance of Logistic Regression should be understood in light of the additive structure of the simulated latent risk score. At the same time, the sigmoid and exponential transformations used to generate the Late-Night Index and the application-switching rate introduce nonlinearity and skewness into the observed features, which may also influence the relative performance of the linear and nonlinear classifiers. Consistent with the across-seed analysis, the small observed differences between algorithms should not be interpreted as evidence of model superiority. No formal pairwise model-superiority testing was performed because algorithm ranking was not a study objective; observed differences were interpreted descriptively in light of the overlapping across-seed performance distributions. Therefore, the algorithm comparison should be viewed as a pipeline-comparison exercise under controlled assumptions, not as evidence that linear models will necessarily perform better in real-world IGD telemetry data.

Within this simulation, the higher performance of the all-feature models is best interpreted as an internal consistency check of the pre-specified signal structure rather than as evidence of the relative value of these features in real-world IGD. Future empirical studies should compare interpretable linear and nonlinear models using real-world longitudinal data, clinically validated outcomes, calibration analyses, and external validation before drawing conclusions about the best modeling approach for practical deployment.

### 4.6. Ethical, Privacy, and Governance Considerations

The proposed architecture was intentionally designed around data minimization. It excludes raw keystrokes, screenshots, screen recordings, message content, browsing content, and other content-level data. Instead, it focuses on aggregated temporal and interactional metadata processed at the client level. This design is central to the translational plausibility of the framework because digital phenotyping in mental health can easily become intrusive if it relies on continuous raw monitoring.

Nevertheless, metadata are not ethically neutral. Even without content capture, behavioral timing, switching patterns, and nocturnal activity may reveal sensitive information about routines, sleep, work, stress, social life, or mental health. Any future implementation would therefore require transparent informed consent, strict data-governance procedures, secure storage, clear limits on secondary use, and user control over monitoring. It would also require careful definition of who can access risk outputs and how those outputs may be used. Future real-world implementation would also need to comply with applicable data-protection frameworks, including requirements for lawful processing, data minimization, transparency, and user rights under the General Data Protection Regulation [24].

This is particularly important in IGD research, where over-pathologization remains a concern [10]. A privacy-preserving AI system should not label high engagement as disorder in isolation. Instead, it should support stepped assessment by identifying patterns that may warrant further psychometric screening, sleep assessment, or clinician review. The ethical value of such a system depends not only on technical privacy protections but also on proportionality, transparency, interpretability, and appropriate clinical governance.

Practical implementation would also need to address sustained user engagement, withdrawal of consent, missing or interrupted telemetry, and heterogeneity across devices, platforms, and software versions.

### 4.7. Implications for Personalized Mental Health Care

The proposed framework is relevant to personalized medicine because it shifts the focus from static, one-time assessment toward individualized behavioral monitoring. In principle, aggregated telemetry could help establish a person-specific baseline and detect meaningful deviations over time. Such deviations could then inform adaptive screening intensity, targeted psychoeducation, sleep-focused assessment, or clinician follow-up.

However, the framework should be understood as a decision-support layer rather than a diagnostic tool. The most appropriate use case would be a stepped-care pathway in which telemetry-based risk signals trigger further assessment rather than directly assign a diagnosis. For example, an elevated risk score could prompt completion of a validated IGD instrument, review of sleep patterns, assessment of functional impairment, or referral to a clinician when clinically appropriate.

This layered approach aligns better with personalized mental health care than a binary diagnostic classifier operating in isolation. It allows behavioral telemetry to contribute objectively logged behavioral information while preserving the central role of validated clinical assessment, patient context, and professional judgment.

### 4.8. Limitations

The main limitation of this study is the use of synthetic data. Although simulation is useful for early-stage development and reproducibility, synthetic profiles cannot capture the full complexity of real gaming behavior, psychiatric comorbidity, social context, game genre, motivation, occupational gaming, cultural variation, sleep schedules, or functional impairment. Therefore, the reported performance metrics demonstrate model behavior under the stated simulation assumptions and should not be interpreted as evidence of real-world generalizability, external validity, or clinical effectiveness.

A second limitation is that the synthetic outcome label was generated from assumptions related to the same features used for prediction. This creates a risk of circularity. The model may partly recover the structure imposed during data generation rather than discover naturally occurring behavioral markers of IGD. To characterize rather than eliminate this limitation, the study incorporated stochastic label noise, cross-validation, model comparison, and across-seed robustness analyses, while interpreting the playtime-only and feature importance analyses as internal consistency checks rather than empirical tests of predictor relevance. However, only real-world validation can determine whether the candidate features generalize beyond the simulation.

A third limitation concerns the cross-sectional structure of the simulation. Personalized medicine requires longitudinal modeling of within-person change, not only between-person classification. Future studies should evaluate whether telemetry features can identify deviations from an individual’s own baseline and whether these deviations predict clinically meaningful outcomes.

A fourth limitation is that the current framework includes only four candidate features. Real-world risk stratification would likely require richer behavioral, contextual, and clinical information, including sleep, mood, stress, functional impairment, comorbid symptoms, and patient-reported outcomes. However, adding more features must be balanced against privacy and interpretability considerations.

Finally, the current study did not evaluate fairness, subgroup performance, or calibration across demographic groups. Future validation studies should examine whether model performance differs by age, gender, cultural background, gaming genre, device type, and baseline gaming intensity. This is essential before any use in real-world screening or care pathways.

### 4.9. Future Directions

Future research should proceed in several stages. First, the proposed simulation framework should be replicated using open code and alternative assumptions about prevalence, feature distributions, label noise, and behavioral heterogeneity. Second, real-world observational studies should collect privacy-preserving telemetry alongside validated IGD scales, sleep measures, functional-impairment indicators, and clinical interviews where feasible. Future studies may also examine whether privacy-preserving telemetry can be combined with complementary AI modalities, including LLM-assisted screening or psychoeducation, while maintaining human oversight, transparent governance, and rigorous clinical validation. Third, longitudinal models should be developed to examine within-person change, followed by external validation across independent populations, games, platforms, and devices. Fourth, calibration and decision-curve analyses should be used to determine whether risk scores provide clinically meaningful information at specific screening thresholds. Finally, implementation studies should evaluate acceptability, consent, governance, and user trust. Consistent with broader concerns about translating healthcare AI from technical development into clinical practice, future validation should evaluate not only discrimination performance but also calibration, workflow integration, safety, acceptability, governance, and clinical utility [25].

The long-term goal should not be an autonomous AI diagnostic system. Rather, the goal should be a privacy-aware, interpretable, and clinically governed digital phenotyping layer that supports personalized monitoring and timely assessment while minimizing surveillance risk.

## 5. Conclusions

This study presents a privacy-preserving, AI-enabled digital phenotyping framework for personalized risk stratification in Internet Gaming Disorder. Using a reproducible synthetic simulation, the proposed pipeline shows that aggregated temporal and interactional telemetry features can be transformed into interpretable simulated risk signals under controlled assumptions. Under the pre-specified synthetic signal structure, the all-feature models showed higher performance than the playtime-only baselines, serving as an internal consistency check of the simulation rather than evidence of their relative value in real-world IGD.

The findings do not establish clinical validity or diagnostic performance. Because the dataset is synthetically generated, the results represent methodological feasibility and internal consistency testing rather than real-world validation. The feature importance results show that the pipeline can recover the pre-specified signal hierarchy encoded in the synthetic generator. They do not identify validated digital markers; rather, they indicate which pre-specified telemetry feature classes may be treated as hypotheses for independent empirical testing.

From a personalized-medicine perspective, the main contribution of this work is methodological. It demonstrates a privacy-aware and interpretable framework for transforming passive behavioral metadata into individualized risk stratification signals while avoiding content-level monitoring. Future research needs to validate this approach using real-world, ethically governed telemetry data and examine whether person-level changes in behavioral patterns can support timely screening, stepped assessment, and personalized mental health care pathways.

## Figures and Tables

**Table 1:** Operational definitions and theoretical relevance of the candidate behavioral telemetry features.

Feature	Variable	Description	Theoretical Relevance
Average session duration	$T_{avg,i}$	Mean duration of uninterrupted gaming sessions	General engagement intensity
Sessions per week	$S_{freq,i}$	Number of sessions initiated within a seven-day period	Repetitive gaming pattern
Late-Night Index	${\mathrm{LNI}}_{i}$	Proportion of gaming time between 00:00 and 06:00	Circadian disruption and persistence despite potential consequences
Application-switching rate	$R_{switch,i}$	Number of application-switching events per active gaming hour	Interactional instability, attentional preoccupation, or difficulty disengaging

**Table 2:** Stratified five-fold cross-validation performance within the training set. Values are mean ± SD across the five validation folds.

(A) Classification Metrics							
Model	Acc.	Bal. Acc.	Sens.	Spec.	PPV	NPV	F1
Random Forest—all features	0.865 ± 0.032	0.808 ± 0.059	0.713 ± 0.120	0.903 ± 0.033	0.652 ± 0.089	0.927 ± 0.028	0.677 ± 0.084
Logistic Regression—all features	0.840 ± 0.033	0.820 ± 0.056	0.788 ± 0.111	0.853 ± 0.034	0.575 ± 0.066	0.942 ± 0.029	0.662 ± 0.070
Gradient Boosting—all features	0.870 ± 0.032	0.769 ± 0.058	0.600 ± 0.107	0.938 ± 0.023	0.707 ± 0.102	0.904 ± 0.024	0.647 ± 0.097
Random Forest—playtime only	0.688 ± 0.023	0.556 ± 0.030	0.338 ± 0.102	0.775 ± 0.050	0.270 ± 0.032	0.825 ± 0.014	0.296 ± 0.058
Logistic Regression—playtime only	0.640 ± 0.060	0.623 ± 0.075	0.594 ± 0.134	0.652 ± 0.067	0.300 ± 0.064	0.866 ± 0.038	0.397 ± 0.084
**(B) Discrimination and Probabilistic Performance**							
**Model**		**ROC-AUC**		**AP**		**Brier**	
Random Forest—all features		0.880 ± 0.027		0.737 ± 0.104		0.101 ± 0.020	
Logistic Regression—all features		0.894 ± 0.027		0.737 ± 0.104		0.117 ± 0.022	
Gradient Boosting—all features		0.860 ± 0.040		0.690 ± 0.129		0.105 ± 0.027	
Random Forest—playtime only		0.591 ± 0.061		0.325 ± 0.086		0.213 ± 0.017	
Logistic Regression—playtime only		0.680 ± 0.060		0.417 ± 0.094		0.225 ± 0.016	

Note: Acc., accuracy; Bal. Acc., balanced accuracy; Sens., sensitivity; Spec., specificity; PPV, positive predictive value; NPV, negative predictive value; F1, F1-score; ROC-AUC, area under the receiver operating characteristic curve; AP, average precision; SD, standard deviation.

**Table 3:** Comparative model performance on the independent hold-out test set for the reference seed-42 realization.

Model	Acc.	Bal. Acc.	Sens.	Spec.	PPV	NPV	F1	ROC-AUC	AP	Brier
Random Forest—all features	0.880	0.850	0.800	0.900	0.667	0.947	0.727	0.909	0.779	0.089
Logistic Regression—all features	0.860	0.856	0.850	0.862	0.607	0.958	0.708	0.920	0.832	0.106
Gradient Boosting—all features	0.900	0.806	0.650	0.962	0.812	0.917	0.722	0.907	0.805	0.073
Random Forest—playtime only	0.725	0.622	0.450	0.794	0.353	0.852	0.396	0.686	0.497	0.189
Logistic Regression—playtime only	0.660	0.656	0.650	0.662	0.325	0.883	0.433	0.724	0.547	0.210

Note: Abbreviations are as defined in Table 2.

**Table 4:** Feature importance analysis for the Random Forest model in the reference seed-42 realization.

Feature	Feature Label	Gini Importance	Permutation Importance, Mean ROC-AUC Decrease	Permutation Importance SD
$R_{switch,i}$	Application-switching rate	0.444	0.0877	0.0267
${\mathrm{LNI}}_{i}$	Late-Night Index	0.405	0.0806	0.0232
$T_{avg,i}$	Average session duration	0.122	0.0273	0.0145
$S_{freq,i}$	Sessions per week	0.029	−0.0008	0.0026

**Table 5:** Random Forest sensitivity analysis across label-noise conditions using reference seed 42.

Label Noise	Acc.	Bal. Acc.	Sens.	Spec.	PPV	F1	ROC-AUC	AP	Brier
0%	0.925	0.944	0.975	0.913	0.736	0.839	0.990	0.966	0.051
5%	0.880	0.850	0.800	0.900	0.667	0.727	0.909	0.779	0.089
10%	0.825	0.731	0.575	0.888	0.561	0.568	0.816	0.570	0.132
15%	0.800	0.716	0.575	0.856	0.500	0.535	0.750	0.481	0.158
20%	0.785	0.650	0.425	0.875	0.459	0.442	0.670	0.392	0.184

Note: The top Gini feature was application-switching rate at all noise levels; the top permutation feature was application-switching rate except at 10% label noise, where it was Late-Night Index.

**Table 6:** Across-seed performance distributions for the evaluated models under the primary 5% label-noise condition across 200 independent synthetic realizations. Values are mean ± SD [empirical 2.5th–97.5th percentile interval].

(A) Classification Metrics							
Model	Acc.	Bal. Acc.	Sens.	Spec.	PPV	NPV	F1
Random Forest—all features	0.882 ± 0.022 [0.835–0.920]	0.837 ± 0.035 [0.775–0.906]	0.761 ± 0.069 [0.650–0.900]	0.912 ± 0.024 [0.863–0.956]	0.689 ± 0.060 [0.574–0.806]	0.939 ± 0.017 [0.911–0.973]	0.721 ± 0.049 [0.629–0.805]
Logistic Regression—all features	0.865 ± 0.024 [0.820–0.915]	0.853 ± 0.029 [0.791–0.903]	0.834 ± 0.056 [0.725–0.926]	0.873 ± 0.029 [0.819–0.925]	0.625 ± 0.055 [0.532–0.745]	0.955 ± 0.014 [0.925–0.980]	0.713 ± 0.043 [0.636–0.800]
Gradient Boosting—all features	0.884 ± 0.020 [0.845–0.920]	0.798 ± 0.038 [0.715–0.875]	0.654 ± 0.077 [0.500–0.825]	0.942 ± 0.019 [0.900–0.975]	0.743 ± 0.065 [0.622–0.867]	0.916 ± 0.017 [0.884–0.955]	0.692 ± 0.056 [0.562–0.800]
Random Forest—playtime only	0.669 ± 0.052 [0.550–0.755]	0.609 ± 0.045 [0.525–0.697]	0.508 ± 0.103 [0.324–0.701]	0.710 ± 0.074 [0.531–0.832]	0.309 ± 0.049 [0.219–0.410]	0.853 ± 0.023 [0.812–0.896]	0.380 ± 0.055 [0.280–0.481]
Logistic Regression—playtime only	0.638 ± 0.033 [0.570–0.705]	0.632 ± 0.042 [0.550–0.716]	0.622 ± 0.079 [0.474–0.800]	0.642 ± 0.038 [0.562–0.713]	0.303 ± 0.033 [0.241–0.377]	0.872 ± 0.024 [0.828–0.922]	0.407 ± 0.044 [0.323–0.496]
**(B) Discrimination and Probabilistic Performance**							
**Model**		**ROC-AUC**		**AP**		**Brier**	
Random Forest—all features		0.894 ± 0.029 [0.839–0.950]		0.751 ± 0.058 [0.633–0.862]		0.094 ± 0.013 [0.070–0.120]	
Logistic Regression—all features		0.904 ± 0.031 [0.835–0.956]		0.773 ± 0.058 [0.655–0.875]		0.109 ± 0.013 [0.085–0.135]	
Gradient Boosting—all features		0.888 ± 0.031 [0.821–0.944]		0.737 ± 0.062 [0.613–0.851]		0.090 ± 0.014 [0.064–0.121]	
Random Forest—playtime only		0.648 ± 0.051 [0.528–0.735]		0.337 ± 0.057 [0.239–0.457]		0.208 ± 0.013 [0.184–0.241]	
Logistic Regression—playtime only		0.681 ± 0.049 [0.589–0.776]		0.379 ± 0.067 [0.272–0.513]		0.224 ± 0.011 [0.205–0.246]	

Note: Bracketed values are empirical 2.5th–97.5th percentile intervals across 200 independent synthetic realizations. Abbreviations are as defined in Table 2.

## Data Availability

The reference seed-42 synthetic dataset, complete revised Python analysis code, and all analysis outputs supporting the reported model evaluation, cross-validation, calibration, feature-correlation, label-noise sensitivity, and across-seed robustness analyses are openly available at https://doi.org/10.5281/zenodo.22018420. No real participant data, patient records, identifiable behavioral logs, or content-level behavioral data were used in this study.

## Supplementary Materials

The following supporting information can be downloaded at: https://www.mdpi.com/article/10.3390/jpm16090447/s1. Table S1: Synthetic data-generation parameters; Table S2: Machine-learning models and baseline comparisons; Table S3: Primary Random Forest performance on the hold-out test set; Table S4: Stratified five-fold cross-validation performance within the training set; Table S5: Sensitivity of all-feature models across label-noise conditions; Table S6: Across-seed performance distributions under the primary 5% label-noise condition (200 synthetic realizations); and Table S7: Pearson correlation matrix of the synthetic telemetry features.

## Abbreviations

The following abbreviations are used in this manuscript:

Acc.	Accuracy
AP	Average precision
AI	Artificial intelligence
Bal. acc.	Balanced accuracy
COVID-19	Coronavirus disease 2019
DSM-5-TR	Diagnostic and Statistical Manual of Mental Disorders, Fifth Edition, Text Revision
F1	F1-score
ICD-11	International Classification of Diseases, Eleventh Revision
IGD	Internet Gaming Disorder
IGDS9-SF	Internet Gaming Disorder Scale–Short Form
I-PACE	Interaction of Person-Affect-Cognition-Execution
LLM	Large language model
LNI	Late-Night Index
NPV	Negative predictive value
PPV	Positive predictive value
ROC-AUC	Area under the receiver operating characteristic curve
SD	Standard deviation
Sens.	Sensitivity
Spec.	Specificity

## Appendix A. Algorithms

### Appendix A.1. Synthetic Data Generation Algorithm

**Algorithm A1.** Synthetic data generation procedure for virtual user profiles.
**Input:** Number of virtual users N; random seed s; elevated-risk prevalence p; label-noise level λ. For the reference analysis, N = 1000, s = 42, p = 0.20, and λ = 0.05. **Output:** Synthetic dataset containing four telemetry features and one binary simulated elevated-risk label. 1. Set the random seed to s (with s = 42 for the reference realization). 2. For each virtual user i = 1, …, N, generate two latent variables: $$I_{i}∼N\left(0,1\right)$$ $$D_{i}∼N\left(0,1\right)$$ where $I_{i}$ represents latent gaming intensity and $D_{i}$ represents latent behavioral dysregulation. 3. Generate average session duration: $$T_{avg,i}=clip\left(1.40+0.75I_{i}+0.25D_{i}+\epsilon_{1i},\;0.20,\;7.50\right)$$ where $\epsilon_{1i}∼N\left(0,0.65^{2}\right)$ 4. Generate sessions per week: $$S_{freq,i}=clip\left[round\left(4.50+2.10I_{i}+0.65D_{i}+\epsilon_{2i},\;1,\;21\right)\right]$$ where $\epsilon_{2i}∼N\left(0,1.80^{2}\right)$ 5. Generate Late-Night Index: $$LNI_{i}=clip\left[\sigma \left(-2.30+0.35I_{i}+1.25D_{i}+\epsilon_{3i}\right),0,1\right]$$ where $\epsilon_{3i}∼N\left(0,0.80^{2}\right)$ and $\sigma \left(x\right)$ is the logistic sigmoid function. 6. Generate application-switching rate: $$R_{switch,i}=clip\left[exp\left(0.60+0.15I_{i}+0.55D_{i}+\epsilon_{4i}\right),\;0.20,\;12.00\right]$$ where $\epsilon_{4i}∼N\left(0,0.35^{2}\right)$ 7. Standardize the four telemetry features across the full synthetic population using z-score transformation for latent risk-score generation. 8. Compute the continuous latent risk score: $$Risk_{i}\;=\;0.15z\left(T_{avg,i}\right)\;+\;0.05z\left(S_{freq,i}\right)\;+\;0.85z\left(LNI_{i}\right)\;+\;1.05z\left(R_{switch,i}\right)\;+\;\eta_{i}$$ where $\eta_{i}∼N\left({0,0.75}^{2}\right)$ 9. Assign the clean simulated elevated-risk label: $$y_{i}^{clean}=\;\left\{\begin{array}{cc} 1, & ifRisk_{i}\geq Q_{0.80}\left(Risk\right)\\ 0, & \mathrm{otherwise} \end{array}\right.$$ where $Q_{0.80}\left(Risk\right)$ is the 80th percentile of the latent risk-score distribution. 10. Introduce balanced stochastic label noise at level λ by flipping equal numbers of positive and negative labels. For the reference condition λ = 0.05 and N = 1000, this corresponds to 25 clean elevated-risk labels changed to lower-risk labels and 25 clean lower-risk labels changed to elevated-risk labels. 11. Return the final synthetic dataset: $${\left\{\left(T_{avg,i},S_{freq,i},{\mathrm{LNI}}_{i},R_{switch,i},y_{i}\right)\right\}}_{i=1}^{1000}$$ where $y_{i}$ is the final noisy simulated elevated-risk label.

### Appendix A.2. Model Evaluation Pipeline

**Algorithm A2.** Machine learning evaluation and robustness pipeline.
**Input:** Synthetic dataset generated by Algorithm A1; full telemetry feature set X; binary simulated outcome y; test proportion = 0.20; reference random seed = 42; label-noise levels =0%, 5%, 10%, 15%, and 20%. **Output:** Hold-out and cross-validated model-performance metrics; ROC and precision–recall curves; Random Forest calibration and feature importance results; feature-correlation matrix; label-noise sensitivity results; and across-seed robustness summaries. 1. Define the full telemetry feature set: $$X_{full}=\left[T_{avg,i},S_{freq,i},\mathrm{LN}I_{i},R_{switch,i}\right]$$ 2. Define the playtime-only baseline feature set: $$X_{playtime}=\left[T_{avg,i}\right]$$ 3. Split the reference seed 42 synthetic dataset into an 80% training set and a 20% independent hold-out test set using stratified sampling so that the lower-risk and elevated-risk class proportions are preserved. 4. Specify the primary all-feature Random Forest classifier using: ○ 100 decision trees; ○ Maximum tree depth = 5; ○ Class weighting set to balanced; ○ Random seed = 42. 5. Specify the all-feature comparator models: ○ Logistic Regression: StandardScaler fitted within the model pipeline, followed by Logistic Regression with class weighting set to balanced, maximum iterations set to 1000, and random seed = 42. ○ Gradient Boosting: 100 estimators, maximum tree depth of 3, and random seed = 42. 6. Specify two playtime-only baseline models using $T_{avg,i}$ as the sole predictor: ○ Random Forest, playtime-only. ○ Logistic Regression, playtime-only. 7. Fit each model using the 80% training set and generate predicted class labels and predicted probabilities for the independent 20% hold-out test set. 8. For each model, calculate hold-out performance using: ○ Accuracy. ○ Balanced accuracy. ○ Sensitivity/recall. ○ Specificity. ○ Precision/positive predictive value. ○ Negative predictive value. ○ F1-score. ○ ROC-AUC. ○ AP. ○ Brier score. Also calculate the confusion matrix for the primary Random Forest model. 9. Within the 80% training set, perform stratified five-fold cross-validation using shuffled folds and random seed = 42. Apply the same pre-specified model configurations in each fold and keep the independent 20% hold-out test set excluded from this procedure. 10. For each model, calculate the same performance metrics within each validation fold and summarize cross-validation performance using the mean and standard deviation across the five folds. Generate pooled out-of-fold predicted probabilities for comparative ROC and precision–recall curves. 11. For the primary Random Forest model, assess probability calibration on the hold-out test set using: ○ The Brier score; ○ A 10-bin calibration curve. No post hoc probability recalibration is applied. 12. For the primary Random Forest model, calculate impurity-based feature importance for all four telemetry features. 13. Calculate permutation feature importance for the primary Random Forest model on the hold-out test set using ROC-AUC as the scoring metric and 50 repeated permutations per feature. 14. Calculate pairwise Pearson correlations among $T_{avg,i},S_{freq,i},{\mathrm{LNI}}_{i},R_{switch,i}$ to characterize the covariance structure relevant to interpretation of the feature importance analyses. 15. Evaluate sensitivity to imperfect simulated ground truth under balanced label-noise conditions 0%, 5%, 10%, 15%, and 20%. For each condition: ○ Regenerate the synthetic dataset using reference seed = 42; ○ Preserve the elevated-risk prevalence at 20% through balanced label flipping; ○ Re-estimate the all-feature Random Forest, Logistic Regression, and Gradient Boosting models; ○ Calculate the same hold-out performance metrics as in Step 8. 16. For the Random Forest model, additionally record the top-ranked feature according to: ○ Impurity-based importance; ○ Permutation importance at each label-noise level to characterize feature-ranking stability. 17. Assess robustness to the particular synthetic realization by repeating the primary 5% label-noise data generation, stratified train–test split, and hold-out evaluation procedure across 200 independent random seeds; for each realization, use the corresponding seed for data generation, train–test splitting, and stochastic model components. 18. For each of the 200 realizations, evaluate: ○ Random Forest, all features; ○ Logistic Regression, all features; ○ Gradient Boosting, all features; ○ Random Forest, playtime-only; and ○ Logistic Regression, playtime-only. 19. For each model and performance metric across the 200 realizations, calculate: ○ Mean; ○ Standard deviation; ○ Empirical 2.5th percentile; and ○ Empirical 97.5th percentile. 20. Compare: ○ All-feature versus playtime-only performance as an internal consistency check of the imposed synthetic signal structure; ○ Performance across the three all-feature algorithms; ○ Degradation across increasing label-noise levels; ○ Variability across independent synthetic realizations. 21. Return all performance tables, cross-validation summaries, ROC and precision–recall curves, calibration outputs, feature importance results, feature-correlation matrix, label-noise sensitivity results, and across-seed robustness summaries.
