# Supplementary material for: AI-Enabled Digital Phenotyping for Personalized Risk Stratification in Internet Gaming Disorder: A Privacy-Preserving Simulation Study
Source: J Pers Med. 2026 Aug 27;16(9):447. doi: 10.3390/jpm16090447 (PMC13608727; doi:10.3390/jpm16090447)
Supplement: Supplementary file 1 [file jpm-16-00447-s001.zip › jpm-4451256-supplementary.pdf]

Supplementary Table S1. Synthetic data-generation parameters.

Component	Specification	Purpose
Sample size	$N = 1{,}000$ virtual user profiles	Simulation-based methodological proof of concept
Reference random seed	42	Reproducible reference realization; additional across-seed robustness evaluated over 200 seeds
Latent intensity	$I_i \sim \mathcal{N}(0,1)$	Generate correlated gaming-engagement patterns
Latent dysregulation	$D_i \sim \mathcal{N}(0,1)$	Generate correlated temporal and interactional dysregulation patterns
Average session duration	$T_{avg,i} = \text{clip}(1.40 + 0.75\ I_i + 0.25\ D_i + \varepsilon_{1i}, 0.20, 7.50)$ $\varepsilon_{1i} \sim \text{N}(0, 0.65^2)$	General engagement intensity
Sessions per week	$S_{freq,i} = \text{clip}(\text{round}(4.50 + 2.10\ I_i + 0.65\ D_i + \varepsilon_{2i}), 1, 21)$ $\varepsilon_{2i} \sim \text{N}(0, 1.80^2)$	Weekly repetition of gaming sessions
Late-Night Index	$LNI_i = \text{clip}[\sigma(-2.30 + 0.35\ I_i + 1.25\ D_i + \varepsilon_{3i}), 0, 1]$ $\varepsilon_{3i} \sim \text{N}(0, 0.80^2)$	Nocturnal gaming and potential circadian disruption
Application-switching rate	$R_{switch,i} = \text{clip}[\exp(0.60 + 0.15\ I_i + 0.55\ D_i + \varepsilon_{4i}), 0.20, 12.00]$ $\varepsilon_{4i} \sim \text{N}(0, 0.35^2)$	Interactional instability or difficulty disengaging
Latent risk score	$0.15\ \text{z}(T_{avg,i}) + 0.05\ \text{z}(S_{freq,i}) + 0.85\ \text{z}(LNI_i) + 1.05\ \text{z}(R_{switch,i}) + \eta_i,\ \ \eta_i \sim \text{N}(0, 0.75^2)$	Controlled simulation of elevated-risk behavioral tendency
Clean class label	Top 20% of latent risk-score distribution coded as elevated risk	Simulated elevated-risk status, not clinical diagnosis
Label noise	5% balanced stochastic label flipping	Approximation of imperfect ground truth while preserving 20% prevalence

Supplementary Table S2. Machine-learning models and baseline comparisons.

Model	Features used	Model specification	Main purpose
Random Forest	All four telemetry features	100 trees; maximum depth = 5; class_weight = balanced; random_state = 42	Primary illustrative model selected a priori; nonlinear structure and feature importance
Logistic Regression	All four telemetry features	StandardScaler within a scikit-learn Pipeline; max_iter = 1000; class_weight = balanced; random_state = 42	Interpretable linear comparator
Gradient Boosting	All four telemetry features	100 estimators; maximum tree depth = 3; random_state = 42	Nonlinear ensemble comparator
Random Forest, playtime-only	Average session duration only	Same Random Forest configuration as the all-feature model	Baseline model using gaming duration alone
Logistic Regression, playtime-only	Average session duration only	Same Logistic Regression pipeline and configuration as the all-feature model	Linear baseline using gaming duration alone

Supplementary Table S3. Primary Random Forest performance on the hold-out test set.

Metric	Value
Test sample size	200
Accuracy	0.880
Balanced accuracy	0.850
Sensitivity / recall	0.800
Specificity	0.900
Precision / positive predictive value	0.667
Negative predictive value	0.947
F1-score	0.727
Area under the receiver operating characteristic curve (ROC-AUC)	0.909
Average precision (AP)	0.779
Brier score	0.089
True negatives	144
False positives	16
False negatives	8
True positives	32

Supplementary Table S4. Stratified five-fold cross-validation performance within the training set.

Values are mean ± SD across five validation folds. The independent 20% hold-out test set was excluded from cross-validation.

Model	Accuracy	Balanced accuracy	Sensitivity	Specificity	PPV	NPV	F1	ROC-AUC	AP	Brier
Random Forest — all features	0.865 ± 0.032	0.808 ± 0.059	0.713 ± 0.120	0.903 ± 0.033	0.652 ± 0.089	0.927 ± 0.028	0.677 ± 0.084	0.880 ± 0.027	0.737 ± 0.104	0.101 ± 0.020
Logistic Regression — all features	0.840 ± 0.033	0.820 ± 0.056	0.788 ± 0.111	0.853 ± 0.034	0.575 ± 0.066	0.942 ± 0.029	0.662 ± 0.070	0.894 ± 0.027	0.737 ± 0.104	0.117 ± 0.022
Gradient Boosting — all features	0.870 ± 0.032	0.769 ± 0.058	0.600 ± 0.107	0.938 ± 0.023	0.707 ± 0.102	0.904 ± 0.024	0.647 ± 0.097	0.860 ± 0.040	0.690 ± 0.129	0.105 ± 0.027
Random Forest — playtime only	0.688 ± 0.023	0.556 ± 0.030	0.338 ± 0.102	0.775 ± 0.050	0.270 ± 0.032	0.825 ± 0.014	0.296 ± 0.058	0.591 ± 0.061	0.325 ± 0.086	0.213 ± 0.017
Logistic Regression — playtime only	0.640 ± 0.060	0.623 ± 0.075	0.594 ± 0.134	0.652 ± 0.067	0.300 ± 0.064	0.866 ± 0.038	0.397 ± 0.084	0.680 ± 0.060	0.417 ± 0.094	0.225 ± 0.016

Supplementary Table S5. Sensitivity of all-feature models across label-noise conditions.

Balanced stochastic label flipping preserved the 20% elevated-risk prevalence at each label-noise level. Values are from the seed-42 reference realization.

Label noise	Model	Acc.	Bal. acc.	Sens.	Spec.	PPV	NPV	F1	ROC-AUC	AP	Brier
0%	Random Forest	0.925	0.944	0.975	0.913	0.736	0.993	0.839	0.990	0.966	0.051
0%	Logistic Regression	0.930	0.956	1.000	0.913	0.741	1.000	0.851	0.997	0.988	0.056
0%	Gradient Boosting	0.970	0.972	0.975	0.969	0.886	0.994	0.929	0.997	0.988	0.024
5%	Random Forest	0.880	0.850	0.800	0.900	0.667	0.947	0.727	0.909	0.779	0.089
5%	Logistic Regression	0.860	0.856	0.850	0.862	0.607	0.958	0.708	0.920	0.832	0.106
5%	Gradient Boosting	0.900	0.806	0.650	0.962	0.812	0.917	0.722	0.907	0.805	0.073
10%	Random Forest	0.825	0.731	0.575	0.888	0.561	0.893	0.568	0.816	0.570	0.132
10%	Logistic Regression	0.820	0.803	0.775	0.831	0.534	0.937	0.633	0.849	0.615	0.151
10%	Gradient Boosting	0.870	0.750	0.550	0.950	0.733	0.894	0.629	0.810	0.634	0.104
15%	Random Forest	0.800	0.716	0.575	0.856	0.500	0.890	0.535	0.750	0.481	0.158
15%	Logistic Regression	0.755	0.716	0.650	0.781	0.426	0.899	0.515	0.738	0.500	0.184
15%	Gradient Boosting	0.830	0.669	0.400	0.938	0.615	0.862	0.485	0.739	0.448	0.137
20%	Random Forest	0.785	0.650	0.425	0.875	0.459	0.859	0.442	0.670	0.392	0.184
20%	Logistic Regression	0.740	0.688	0.600	0.775	0.400	0.886	0.480	0.700	0.450	0.208
20%	Gradient Boosting	0.785	0.575	0.225	0.925	0.429	0.827	0.295	0.673	0.391	0.155

Supplementary Table S6. Across-seed performance distributions under the primary 5% label-noise condition (200 synthetic realizations).

Values are mean ± SD [empirical 2.5th–97.5th percentile interval] across 200 independent synthetic realizations. Seed 42 remains the reference realization reported in the main manuscript.

Model	Acc.	Bal. acc.	Sens.	Spec.	PPV	NPV	F1	ROC-AUC	AP	Brier
Random Forest — all features	0.882 ± 0.022 [0.835–0.920]	0.837 ± 0.035 [0.775–0.906]	0.761 ± 0.069 [0.650–0.900]	0.912 ± 0.024 [0.863–0.956]	0.689 ± 0.060 [0.574–0.806]	0.939 ± 0.017 [0.911–0.973]	0.721 ± 0.049 [0.629–0.805]	0.894 ± 0.029 [0.839–0.950]	0.751 ± 0.058 [0.633–0.862]	0.094 ± 0.013 [0.070–0.120]
Logistic Regression — all features	0.865 ± 0.024 [0.820–0.915]	0.853 ± 0.029 [0.791–0.903]	0.834 ± 0.056 [0.725–0.926]	0.873 ± 0.029 [0.819–0.925]	0.625 ± 0.055 [0.532–0.745]	0.955 ± 0.014 [0.925–0.980]	0.713 ± 0.043 [0.636–0.800]	0.904 ± 0.031 [0.835–0.956]	0.773 ± 0.058 [0.655–0.875]	0.109 ± 0.013 [0.085–0.135]
Gradient Boosting — all features	0.884 ± 0.020 [0.845–0.920]	0.798 ± 0.038 [0.715–0.875]	0.654 ± 0.077 [0.500–0.825]	0.942 ± 0.019 [0.900–0.975]	0.743 ± 0.065 [0.622–0.867]	0.916 ± 0.017 [0.884–0.955]	0.692 ± 0.056 [0.562–0.800]	0.888 ± 0.031 [0.821–0.944]	0.737 ± 0.062 [0.613–0.851]	0.090 ± 0.014 [0.064–0.121]
Random Forest — playtime only	0.669 ± 0.052 [0.550–0.755]	0.609 ± 0.045 [0.525–0.697]	0.508 ± 0.103 [0.324–0.701]	0.710 ± 0.074 [0.531–0.832]	0.309 ± 0.049 [0.219–0.410]	0.853 ± 0.023 [0.812–0.896]	0.380 ± 0.055 [0.280–0.481]	0.648 ± 0.051 [0.528–0.735]	0.337 ± 0.057 [0.239–0.457]	0.208 ± 0.013 [0.184–0.241]
Logistic Regression — playtime only	0.638 ± 0.033 [0.570–0.705]	0.632 ± 0.042 [0.550–0.716]	0.622 ± 0.079 [0.474–0.800]	0.642 ± 0.038 [0.562–0.713]	0.303 ± 0.033 [0.241–0.377]	0.872 ± 0.024 [0.828–0.922]	0.407 ± 0.044 [0.323–0.496]	0.681 ± 0.049 [0.589–0.776]	0.379 ± 0.067 [0.272–0.513]	0.224 ± 0.011 [0.205–0.246]

Note: Acc., accuracy; Bal. acc., balanced accuracy; Sens., sensitivity; Spec., specificity; PPV, positive predictive value; NPV, negative predictive value; F1, F1-score; ROC-AUC, area under the receiver operating characteristic curve; AP, average precision; SD, standard deviation.

Supplementary Table S7. Pearson correlation matrix of the synthetic telemetry features.

Correlations are calculated for the 1,000-profile seed-42 reference dataset.

Feature	Average session duration	Sessions per week	Late-Night Index	Application-switching rate
Average session duration	1.000	0.596	0.329	0.291
Sessions per week	0.596	1.000	0.282	0.299
Late-Night Index	0.329	0.282	1.000	0.672
Application-switching rate	0.291	0.299	0.672	1.000
